# Liquor Soda Tartras

**Published:** 1860-11

**Authors:** 


### Liquor Sodæ Tartras.

From an article by Prof. J. Lawrence Smith, in the American Journal of Pharmacy, copied into the Journal of Materia Medica, we learn the formula adopted in the preparation of this solution by T. E. Jenkins & Co., and at the Louisville Chemical Works. It is as follows:

Sal Soda, 21 lbs. 14 oz. avoirdupois.
Tartaric Acid, 15 lbs.
Sugar (white), 24 lbs.
Water, to make, 25 gallons.

It is then put into strong twelve ounce bottles, and thirty-five grains of bi-carbonate of soda added to each bottle, and immediately corked and fastened by twine or wire.

Prof. Smith speaks of it as decidedly preferable to citrate of magnesia. While we consider the solution of citrate of magnesia one of the very best aperients, there are objections to the preparation, particularly the liability to decomposition. The solution of tartrate of soda, it is said, is free from this difficulty, and at the same time possesses the aperient property to the full extent, and is even more prompt and regular in its action.

